# The effects of food craving and desire thinking on states of motivational challenge and threat and their physiological indices

**DOI:** 10.1007/s40519-018-0525-y

**Published:** 2018-06-21

**Authors:** Daniel Frings, Guleser Eskisan, Gabriele Caselli, Ian P. Albery, Antony C. Moss, Marcantonio M. Spada

**Affiliations:** 1 Centre for Addictive Behaviours Research, School of Applied Sciences, London South Bank University, 103 Borough Road, London, SE1 0AA UK; 2 Studi Cognitivi, Milan, Italy; 3 Sigmund Freud University, Milan, Italy; 4 Sigmund Freud University, Vienna, Austria

**Keywords:** Challenge, Threat, Craving, Desire, Cortisol, Cardiovascular, Adrenaline

## Abstract

**Purpose:**

Food craving has been shown to induce states of psychological challenge, indexed by increases in adrenaline but not cortisol production. The study aimed to test the relationship between challenge and (1) desire thinking (the active processing of the pleasant consequences of achieving a desired target and planning how to do so) and (2) craving.

**Methods:**

Participants (*N* = 61) self-reported their levels of craving and desire thinking. They were then presented with situations in which their craving would be fulfilled or not via a false feedback practice task (a wordsearch task). During this period psycho-physiological measures of challenge and threat were taken.

**Results:**

Higher levels of craving were linked to challenge only when the craved object was likely to be obtained. Whilst anticipating reward fulfillment, higher levels of craving were linked to higher levels of desire thinking. In turn, higher levels of desire thinking were related to lower levels of challenge. In contrast, during the processes of reward fulfillment, desire thinking was linked to increased challenge (i.e., a positive indirect effect).

**Conclusions:**

Craving is linked to increased levels of psychological challenge when the object of the craving can be obtained, but it is unrelated to craving when it is not. The research also highlights the importance of desire thinking as an important, but complex, mediator in the relationship between craving and motivational states: desire thinking inhibited challenge when anticipating craving fulfillment, but encouraging it during the process of fulfillment itself.

**Level of evidence:**

I: Evidence obtained from at least one properly designed randomized controlled trial.

## Introduction

Craving is defined as a powerful subjective experience that motivates people to achieve a target [1]. Craving has also been shown to affect people’s capacity for behavioral restraint when dieting [2] and discontinuing engagement in addictive behaviors [3]. However, there remains a need to refine our understanding of the underlying psychological mechanism(s) which may underpin it. The current study addresses this important question by examining how a newly identified psychological construct—desire thinking—interacts with craving to affect both psychological motivation and the neuroendocrine system.

Craving has long been identified as an important contributor to behavioral loss of control and is considered a key area of treatment focus for addictive behaviors [4]. Research evidence has demonstrated that the experience of craving is qualitatively similar across a range of targets, including alcohol, food, soft drinks and tobacco [5–7]. Craving has also been shown to be a major risk factor in triggering relapse [8] and in predicting generally worse outcomes in treatment for substance abuse [9, 10]. Tackling craving is highly clinically relevant-treatment approaches that focus on the regulation of urges have proven to be effective in reducing rates of relapse in various behavioral domains [4, 11].

The elaborated intrusion (EI) theory of desire [6, 12, 13] purports that the experience of craving may arise from the combination of automatic and voluntary cognitive processes. In the EI theory, automatic processes are described as associations which encapsulate information about a desired target or activity (e.g., its positive consequences) and which spontaneously intrude into consciousness leading to the activation of craving. Voluntary processes are described as the activation of forms of cognitive elaboration that lead to the escalation and persistence of craving [13].

Research undertaken over the last decade has shown that a distinctive form of cognitive elaboration, which has been termed ‘desire thinking’ [14, 15], may be closely associated with the intensification of craving. Desire thinking has been described as a conscious and voluntary trait-like tendency characterized by the prefiguring of images, information and memories about positive target-related experience [14]. This prefiguration activity is characterized by the active and controlled processing of the pleasant consequences of achieving a desired target, as well as reviewing good reasons for reaching it, and mentally planning how to do so [14–17]. A second facet of desire thinking is verbal perseveration. This refers to prolonged self-talk regarding presumed worthwhile reasons for engaging in target-related activities and their achievement.

A large body of research indicates that thinking about a desired target is closely associated both to the intensity of craving and physiological changes similar to the direct experience of craving [18–21]. Several studies have confirmed that desire thinking is associated to craving in individuals presenting with alcohol abuse, nicotine dependence and problematic gambling [14]. Desire thinking has also been found to predict craving across a range of addictive behaviors in both community and clinical samples [22–25].

These findings have been confirmed with both longitudinal and experimental designs. First, desire thinking has been found to prospectively predict craving and binge drinking in a non-clinical sample [15]. Secondly, the experimental induction of desire thinking in a sample of patients with alcohol use disorder led to a significant increase in distress and urge to use alcohol when compared to a behavioral assessment test and a distraction task [26]. Finally, an evaluation of psychometric measures of desire thinking and craving has demonstrated only a moderate correlation between the two constructs supporting the distinction between craving and desire thinking [23] and was shown to play a confounder role between mindfulness and alcohol-related craving in a cross-cultural study [27].

### Craving, desire thinking, motivation states and neuroendocrine responses

Craving and desire thinking may affect peoples’ motivational states directly, which in turn will affect individuals’ approach to motivated performance situations and related neuroendocrine system responses. One motivational approach which is of particular relevance is the bio-psychosocial model of challenge and threat (BPSM [28, 29]). BPSM argues that, when in performance motivated situations (i.e., when we are motivated to perform to achieve a goal), we make appraisals concerning the balance of demands (i.e., task difficulty, risk, uncertainty, required effort, etc.) and resources (ability, support from others, etc.). If demands outweigh resources, a state of threat is experienced. If resources outweigh demands, a state of challenge is experienced. Evidence strongly suggests that challenge leads to a focus on gains, better task performance and is linked to positive affect [28, 30–33].

Challenge is linked with increased sympathetic-adrenal-medullary (SAM) activation, leading to adrenaline (epinephrine) production. Adrenaline in turn leads to increased heart rate (HR), increased force of left ventricle ejection (ventricular contractility [VC], linked to the length of the ejection, the left ventricular ejection time, [LVET]). VC is itself calculated using the heart’s pre-ejection period (PEP, the time between the ‘Q’ point of the QRS wave and the ejection). Adrenaline also results in increased cardiac output (CO, the amount of blood ejected from the heart in a given time) and increased vasodilatation (i.e., decreased total peripheral resistance; TPR). In a threat state, simultaneous activation of the hypothalamic–pituitary-adrenal axis occurs. Resultant cortisol production inhibits vasodilatation leading to (relatively) increased TPR. Challenge and threat states are calculated using the above indexes such that they are conceptualized as two ends of a bi-polar continuum. Thus, higher levels of challenge can also be interpreted as lower rates of threat (and vice-versa) [26–30]. Links between these cardiac indexes and cognitive/behavioral outcomes has been empirically observed in a variety of performance situations [29, 34–37].

### Challenge/threat and craving

Recently, some research has suggested the importance of the goal acting as a resource during such appraisals. Frings et al., demonstrated this in the context of food (specifically chocolate) craving [38]. They measured levels of craving for chocolate amongst participants. These participants completed two wordsearch tasks (which they rated as only moderately difficult). The first of these was a ‘practice’. In the second, participants believed the amount of chocolate they would be given as part of their participant payment was contingent on performance. Higher levels of craving were linked to greater challenge. Frings et al., suggest that craving may increase the value of the goal and, as such, may act as a resource. One possible caveat to this is that such appraisals are only likely to occur if the fulfillment seems likely. Where fulfillment is unlikely (for instance, when prior feedback suggests performance will be insufficient) a reverse pattern would be expected—the goal is important (as it is craved) but unobtainable (thus acting as a demand). Under such conditions, it would be predicted that greater craving links to greater threat.

**Fig. 1:**
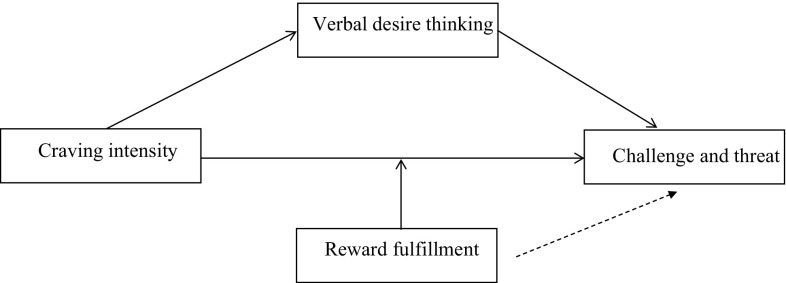
The mediation and moderation model tested. Coefficient values for each relationship can be found in Table 3

As discussed above, craving does not occur in psychological isolation—it is often associated with desire thinking. To the extent thinking about a desired target has been shown to act as a high cognitive load [39], it should also act as a demand during resource evaluations. This could be the case particularly for the verbal component of desire thinking because the well-known impact of verbal repetitive thinking on concentration in cognitive tasks [40]. As such, we predict that the verbal preservation component of desire thinking should also act as a situational demand (as it increases task difficulty, a key demand [29]) with higher levels of verbal desire thinking being associated with lower levels of challenge (higher levels of threat). Taken together, this set of relationships suggests that craving may have two simultaneous, and oppositional, effects when there is an expectation that craving will be fulfilled (see Fig. 1). Specifically, craving will directly increase challenge (as it increases the value of a reward). It should also increase desire thinking. This is particularly likely to be the case when anticipating an attempt to fulfill a craving (as opposed to actually attempting to fulfill it) because desire thinking can be activated as a strategy to: (1) sustain the wait by increasing positive sensations through virtual imagery; (2) improve self-control; (3) explore the best reasons to give way to the temptation; and (4) plan how to organize attempts to fulfill the target [17].

### Aims and hypotheses

In the current study, we replicated Frings et al., by linking levels of craving to subsequent challenge and extended this work by manipulating participants’ expectation that their cravings would be fulfilled (or not). We thus aimed to test a number of hypotheses around the relationships between craving, desire thinking, and challenge/threat states. Specifically, we hypothesized that only under conditions of reward fulfillment will higher levels of craving lead to challenge. The current study also tested the relationships between craving and desire thinking, and their combined effects on challenge/threat states. It was hypothesized that levels of craving would be positively linked to both challenge states and levels of desire thinking. However, as desire thinking increases cognitive load (a demand), it should itself be negatively linked to states of challenge. Thus, we also predicted that craving will encourage states of challenge directly, but simultaneously encouraging threat (i.e., decreased challenge) via its effects on desire thinking.

## Method

### Participants

Sixty-one^1^ participants (53 female, 8 male) were recruited from a modern UK university. The sample’s ages ranged from 19 to 44 years old (*M* = 23.87, SD = 5.24). Inclusion criteria included being prepared to undergo the physiological measures taken (see “Measures” below) and liking chocolate. Exclusion criteria for the study were (1) being under 18 years of age and/or (2) self-report of any of the following; an unstable medical condition, neurological disorder or any history of seizure or epilepsy, pace-maker or similar device use, recent or current upper respiratory tract infection or a fever, having ingested an alcoholic beverage within 12 h of reporting to the laboratory, drug use (prescription, investigational or recreational), being allergic to adhesive tape or alcohol swabs, being unable for any reason consume chocolate and/or find tests (e.g., wordsearches for prizes) overly anxiety provoking.

### Design

An experimental design was used, with one between participant factor (Reward fulfillment: Fulfillment vs. Non-fulfillment). Participants were assigned to condition randomly (Table 1 details sample sizes by condition).

**Table 1:** Sample characteristics across condition

	Reward condition		
	Fulfillment (*n* = 26)	Non-fulfillment (*n* = 25)	Between condition comparison
Gender (male/female)	(3/23)	(5/20)	*x*^2^(2) = 0.01, *p* = .75
Age (years)	25.35 (7.66)	22.77 (2.80)	*t*(59) = 1.84, *p* = .07
Desire thinking	2.55 (0.72)	2.41 (0.60)	*t*(59) = 0.79, *p* = .43
Craving	3.49 (2.43)	4.32 (2.08)	*t*(59) = 1.45, *p* = .15
Body mass index (BMI)	23.37 (4.45)	22.84 (2.14)	*t*(51), = 0.91, *p* = .37

BMI reflects kg/m^2^

### Materials

*Desire thinking* Verbal preservation of desire thinking was measured using the verbal preservation subscale of the Desire Thinking Questionnaire [23]. This measures the tendency towards prolonged and perseverative self-talk regarding a desired activity and its achievement. The original validated scale is in Italian—here items were translated into British English (and back-translated to ensure accuracy). The subscale consisted of five items (e.g., ‘If I did not practice the desired activity for a long time, I would think about it continuously’). All items were rated on a 4-point Likert-type scale (1 = ‘*Almost never*’, 2 = ‘*Sometimes*’, 3 = ‘*Often*’, 4 = ‘*Almost always*’). The reliability of this scale was good—Cronbach’s *α* = 0.73.

*Craving intensity* Craving intensity was measured using an adapted craving intensity subscale of the Craving Experience (Strength) Questionnaire (CEQ-S [42]). Participants were asked to answer each question ‘When thinking about eating chocolate...’ and then to subsequently rate their current craving experience. The original scale was adapted such that each item was prefaced by ‘Right now’ (e.g., ‘Right now, how much do you want it?’) and each was scored on an 11-point Likert-type scale (anchored at 0, *Not at all* and 10, *Extremely*). The reliability of this scale was good—αs = 0.86.

*Word searches* Two 20 × 20 grid wordsearches were presented to each participant. In the reward fulfillment condition, the first wordsearch included a list of 15 target words, all of which could be found in the grid. In the non-fulfillment condition the same list of 15 targets was presented, only three of which were actually included in the grid. In both conditions the first wordsearch was presented as a practice activity and instructions indicated that after the second wordsearch they would be rewarded with a full bar of chocolate for finding 5 words, and a full bar for finding all 15. All participants then completed a new 20 × 20 grid wordsearch in the task phase, containing 15 new words, all of which were actually included in the grid.

*Difficulty* As a manipulation check, the difficulty of each wordsearch was rated on a Likert scale (1 ‘*Very easy*’ to 7 ‘*Very difficult*’) with the following item ‘On the whole I found this [first/second] word search…’.

*Challenge and threat measures* Measures of challenge and threat were taken using impedance cardiography (ICG), electrocardiography (ECG) and continuous blood pressure monitoring in line with previous practice [38]. Indexes derived from these measures included cardiac output (CO), heart rate (HR), pre-ejection period (PEP), left ventricular ejection time (LVET) and total peripheral resistance (TPR). Data from the last 2 min of the baseline phase, and the first 2 min of the anticipatory and task phases were used. These measures were combined to generate a single challenge/threat index for each sets of reactivity (see previous work for methodological specifics [28, 38]). Higher values indicate relative states of challenge, whilst lower scores indicate relative states of threat (with scores above and below 0 indicating relative challenge/threat, respectively).

### Procedure

Once they had consented, participants were asked to rest whilst the baseline physiological measures were undertaken. Participants then completed the craving and desire questionnaire. Participants then completed the two wordsearches. During each of these, physiological measures were taken (comprising the anticipatory and task phase measures, respectively). Difficulty measures were taken immediately after each wordsearch was completed. Upon study completion, all participants completed a funneled debriefing [43] to test for suspicion of the manipulation or the purpose of the study. None were excluded as a result of this process. All participants received a full bar of chocolate and their choice of research participation credits or an online-shopping voucher (worth £10).

## Results

*Randomization check* The gender split within each condition can be seen in Table 1, alongside within condition means for age, desire thinking, craving and body mass index (BMI, kg/m^2^). In summary, the randomization was successful on all these variables.

*Challenge/threat and performance* Across conditions, higher levels of challenge in the anticipatory phase were related to more words being identified in the anticipatory phase, *r*(*n* = 56) = 0.37, *p* = .006. No other correlations between wordsearch score and challenge/threat indexes approached significance (*p*s > .19). In summary, challenge was linked to performance only in the anticipatory phase.

*Physiological indices and task engagement* Full physiological data were not recorded (due to equipment failure) for five participants (*n* = 1 in the fulfillment condition and *n* = 4 in the non-fulfillment condition). These participants were excluded from this and subsequent analysis containing these indices (leaving a final *n* = 56). Mean values for each cardiovascular index at each phase can be seen in Table 2. Within-subject *t* tests revealed that HR increased between the baseline and anticipatory phases [*t*(55) = 2.90, *p* = .005] and between baseline and task phase [*t*(55) = 2.16, *p* = .035]. Similarly, PEP scores increased between base and anticipatory phases [*t*(55) = 2.67, *p* = .01] and between base and task phases [*t*(55) = 3.40, *p* < .001]. In summary, increases in these indexes suggest that participants experienced task engagement.

**Table 2:** Mean cardiac output values by study phase

	Phase		
	Base	Anticipatory	Task
Heart rate (HR)	77.56 (9.99)	80.53 (11.11)	79.87 (10.86)
Pre-ejection period (PEP)	0.10 (0.02)	0.11 (0.04)	0.11 (0.04)
Left ventricular ejection time (LVET)	0.32 (0.05)	0.30 (0.04)	0.29 (0.05)
Cardiac output (CO)	4.89 (2.01)	4.83 (1.61)	4.55 (1.40)
Total peripheral resistance (TPR)	1644.08 (306.59)	1723.14 (445.49)	1811.51 (434.81)

Heart rate HR is reported in beats per minute, CO in liters per minute, PEP and LVET in tenths of a second, and TPR in dynes s/cm^5^

### Effects of fulfillment condition

When examining the baseline and the anticipatory phase index, no differences were observed between the fulfillment condition (*n* = 25, *M* = 0.15, SD = 2.04) relative to the non-fulfillment condition (*n* = 31, *M* = − 0.12, SD = 1.29), *t*(54) = 0.62, *p* = .539. For baseline to task phase reactivity, participants were relatively challenged in the fulfillment condition (*M* = 0.68, SD = 1.53) relative to the non-fulfillment (*M* = − 0.55, SD = 1.62) condition, *t*(54) = 2.89, *p* = .006. In summary, there was no difference between fulfillment conditions in challenge/threat reactivity during the anticipatory phase. However, in the task phase, fulfillment condition participants were more challenged than non-fulfillment participants.

### Moderation and mediation analysis

To test the hypotheses that (a) craving would lead to challenge only when fulfillment seems possible and (b) that the effect of craving should also operate through desire thinking, two moderation and mediation models were constructed and tested using the Hayes PROCESS macro (Model 5) [44].

In one model, the outcome variable was the challenge threat index calculated between the baseline and the anticipatory phase. In the second, it was the index calculated between the baseline and task phases. This variable was predicted by craving intensity, with desire thinking included as a mediator (see Fig. 1). The moderation influence of task fulfillment condition was tested on the direct (but not the indirect) effect of craving on challenge and threat. Models consisted of 5000 bootstrapped samples. Confidence intervals at 95% are reported.

*Anticipatory phase model* Coefficient values (and overall model statistics and interaction terms) can be found in Table 3. The overall model significantly predicted challenge/threat indexes. Higher levels of craving were linked with higher levels of desire thinking, and increased challenge. This latter relationship was moderated by fulfillment condition—when the anticipatory word search was achievable, a positive relationship between craving and challenge threat scores was observed. This effect was not present when task difficulty was high. Desire thinking was negatively related to challenge/threat (i.e., greater levels of desire thinking lead to lower levels of challenges/higher threat). The indirect effect of craving via desire thinking was negative and significant. In summary, a suppression effect was present—craving was related to challenge via its direct effect (when it was anticipated the craving could be fulfilled), but it also (to a lesser extent) related to threat via its influence on desire thinking.

**Table 3:** Coefficients for mediation and moderation analysis

Phase	Coefficient	Value (standard error)	*T*	Lower CI	Upper CI
Anticipatory^1^	Craving–desire thinking	0.10 (0.04)*	2.51	0.02	0.18
	Desire thinking–challenge/threat	− 0.72 (1.22)*	2.51	− 1.30	− 0.14
	Craving–challenge/threat (indirect)	− 0.07 (0.04)	N/A	− 0.18	− 0.02
	Craving–challenge/threat (direct)	1.39 (0.29)*	5.23	0.86	1.92
	Reward fulfillment–challenge/threat	2.12 (0.75)*	2.84	0.62	3.62
	Moderating effect of reward fulfillment	− 0.69 (0.17)	4.09	− 1.03	− 0.35
	Craving–challenge/threat (direct, fulfillment condition)	0.70 (0.12)*	5.82	0.46	0.94
	Craving–challenge/threat (direct, no fulfillment condition)	0.01 (0.12)	0.07	− 0.24	0.26
Task^2^	Craving–desire thinking	0.10 (0.04)*	2.51	0.02	0.18
	Desire thinking–challenge/threat	0.63 (0.29)*	2.12	0.03	1.22
	Craving–challenge/threat (indirect)	0.06 (0.03)	N/A	0.02	0.14
	Craving–challenge/threat (direct)	0.13 (0.27)	0.49	− 0.41	0.68
	Reward fulfillment–challenge/threat	− 1.77 (0.77)^*^	2.31	− 3.31	− 0.23
	Moderating effect of reward fulfillment	0.11(0.17)	0.63	− 0.24	0.46

Model tested is specified in Fig. 1: ^1^ = total model: *R*^2^ = 0.41, *F*(4, 51) = 8.69, *p* ≤ .001. ^2^Total model: *R*^2^ = 0.40, *F*(4,51) = 8.38, *p* < .001

*Significant at *p* < .05.

*Task phase model* In this model, craving had no direct effect upon challenge and threat, but did positively relate to desire thinking. Desire thinking had a positive relationship with levels of challenge (in contrast to the previous model) and the indirect effect was positive and significant. The interaction term between condition and craving did not approach significance. In summary, this model showed a negative indirect relationship between craving and challenge states, driven by the negative relationship between desire thinking and challenge.

## Discussion

Craving is a key concept in addiction as it is relevant to risk of relapse and treatment effectiveness [3]. However, little research has addressed the underlying processes which may affect its escalation, or its effects on motivational states and the neuroendocrine system. The current study aimed to explore how one particular trait, desire thinking, would relate to craving. It also aimed to test how these constructs would, in combination, affect motivational states and subsequent neuroendocrine system responses.

Previous work reveals that higher levels of craving relate to higher levels of challenge (associated with higher levels of adrenaline but not cortisol), when the craving was likely to be realized [38]. The current study replicated this effect, and extended it by manipulating whether or not the craving would be fulfilled. When craving could not be fulfilled, craving was unrelated to challenge/threat states. This suggests that the ability of craving to act as a resource through increasing the value of a reward is only present when people believe they are likely to attain it.

The current study also examined the relationship between craving and challenge/threat responses with a related trait, desire thinking. As desire thinking has been shown to be associated to levels of craving and maladaptive behaviors across a wide range of addictive disorders [17], it was predicted that it would be positively related to levels of craving. This was observed both during the anticipatory phase and the task phase in the current study. As engaging in desire thinking requires significant cognitive effort, it was hypothesized that desire thinking would act as a demand, resulting in lower levels of challenge.

The final relationship the current study predicted was a suppression mediation between craving, desire thinking and levels of challenge/threat. Specifically, higher levels of craving were expected to lead to both higher levels of desire thinking and higher levels of challenge. Higher levels of desire thinking itself, however, were expected to lead to lower levels of challenge. In the anticipatory phase, both a positive direct and a negative indirect relationship were observed between craving and challenge. Contrary to expectations, this same effect was not observed in the task phase. During this period of the study, the effects of desire thinking reversed such that higher levels of desire thinking were linked with higher levels of *challenge*. This reveals that desire thinking had two different and opposite impacts in the relationship between craving and motivational challenge/threat states in the anticipatory phase and the attempting phase. It is plausible to assume that, independently from desire thinking features, goals associated to its activation may play a crucial role in the relationship between craving and motivational states [17]. In particular, in the anticipatory phase, desire thinking may be activated as a strategy for achieving an internal self-regulatory goal (e.g., to cope with a here-and-now feeling of desire). Recent research has shown that individuals with addictive behaviors report adopting desire thinking in order to reach behavioral self-control or to effectively cope with negative feelings like the sense of deprivation [14, 45]. Thus, desire thinking may lead to an immediate reduction in challenge states associated with craving (i.e., have a suppressive effect) because of cognitive demands associated with negative affect. In addition, desire thinking may increase the importance of target-related goals by fixing attention in the present, making salient task demands [22, 23]. Thus, desire thinking during anticipation may reduce challenge by biasing the resources/demands balance via the generation of cognitive conflict between increasingly important goals and increasing cognitive demands for achieving the goal. This conflict may maintain states of psychological threat. In contrast, during the task phase, desire thinking may facilitate the increase of challenge states by self-motivational thinking that can highlight the importance of the goal and a direct elaboration of plan of actions—a more future-orientated response [38].

Alongside the implications for desire thinking, another important implication of the current research is that behaviors which have a craving element to them can be understood via their effects on motivation—as measured via physiological indices of challenge and threat. In particular, we highlight that craving may indirectly affect the neuroendocrine system. In the current study, the effects of craving (when it could be fulfilled) were linked to states of challenge (indexed by cardiac responses reflecting increased adrenaline production, with no accompanied increase in cortisol production). In contrast, desire thinking was linked to threat (indexed by cardiac responses reflecting both increased adrenaline and cortisol production). Long-term overproduction of cortisol, for instance due to extended states of threat, can impact negatively on health—being linked with increased risk of outcomes such as Type 2 diabetes and heart disease [46, 47]. Furthermore, in the anticipatory phase, desire thinking may lead to a threat state increasing the probability of engaging in problematic behavior. In the task phase, desire thinking may increase goal salience and ‘on-line’ conviction in permissive beliefs. These cognitive processes may compete (or override) other more self-inhibitory related information (e.g., good reasons to stop achieving target). This is in line with existing research on the role of desire thinking in individuals with addictive behaviors [17, 26, 27].

The current study has a number of limitations. First, the design did not account for the valence of chocolate eating as a behavior. For some, eating chocolate may be an unambiguously pleasurable activity. For others (e.g., dieters) it may in itself be negatively or ambiguously valenced. For the latter, the taboo temptation of chocolate may act as a demand and subsequently lead to more threat. Although this source of error variance was not controlled for in the current study, independent effects of craving and desire thinking were still observed. Future work could explore this further by comparing people who crave chocolate and wish to act on the craving against those who crave but wish to avoid acting on it. An interesting prediction could be made that both craving and desire thinking will lead to threat when people wish to avoid fulfillment. A second limitation (shared with much BPSM research) is that as challenge and threat are conceived as ends of a bi-polar continuum, firm statements about whether a factor, for instance desire thinking, is associated with an *increase* in challenge or a *decrease* in threat cannot be made.

Finally, the current work assumed a similar relationship between craving and desire thinking when levels of craving are higher or lower. This may not be the case. For instance, amongst those low in craving, a small increase in craving may not lead to an increase in desire thinking (perhaps until some threshold is reached). Alternatively, for those low in craving, even a small increase may lead to a large increase in desire thinking (as this state is not present at all previously). This variance is currently contained in the error variance in our statistical models, and could not be tested with the present sample size. However, despite this, it is worth noticing that a significant effect was observed for the craving–desire relationship.

In summary, the findings demonstrate that craving is linked to increased levels of psychological challenge when the object of the craving can be obtained, but that it is unrelated to craving when it is not. Craving was also shown to be linked to increased desire thinking. Desire thinking was linked to increased threat when anticipating the goal attainment (such that craving also had an indirect threat effect), but increased challenge during the actual attempt itself. This suggests that desire thinking can act as both a demand and resource in challenge/threat appraisals, and also may act as a potential target for interventions. Future research exploring these findings, and the direction of any supposed causal effects, may have beneficial implications for the development of therapeutic approaches such as metacognitive therapy.

## Footnotes

1 Sufficient to detect interactions with effect sizes of Cohen’s *f* = 0.20 (i.e. a small to medium effect [41]) in repeated measures ANOVA with two groups and measurement points, α = 0.05 and power of 0.95).
